# A Comparison of COVID-19 Stigma and AIDS Stigma During the COVID-19 Pandemic: A Cross-Sectional Study in China

**DOI:** 10.3389/fpsyt.2021.782501

**Published:** 2021-12-01

**Authors:** Manyun Li, Jiang Long, Xuyi Wang, Yanhui Liao, Yueheng Liu, Yuzhu Hao, Qiuxia Wu, Yanan Zhou, Yingying Wang, Yunfei Wang, Qianjin Wang, Yuejiao Ma, Shubao Chen, Tieqiao Liu

**Affiliations:** ^1^Department of Psychiatry, and National Clinical Research Center for Mental Disorders, The Second Xiangya Hospital of Central South University, Changsha, China; ^2^Shanghai Mental Health Center, Shanghai Jiao Tong University School of Medicine, Shanghai, China; ^3^Department of Psychiatry, Sir Run Run Shaw Hospital, School of Medicine, Zhejiang University, Hangzhou, China; ^4^Department of Psychiatry, Hunan Brain Hospital (Hunan Second People's Hospital), Changsha, China

**Keywords:** COVID-19, AIDS, stigma, physical avoidance, public panic

## Abstract

**Objective:** To understand the current situation of stigmatizing attitudes toward Coronavirus Disease 2019 (COVID-19) in China and compare it with acquired immunodeficiency syndrome (AIDS).

**Methods:** Convenient sampling and vignette-based methods were used to recruit participants on WeChat. A demographic form and adopted stigma scale were used to collect participants' demographic information and stigmatizing attitudes toward COVID-19 and AIDS.

**Results:** A total of 13,994 questionnaires were included in this study. A high portion of participants tend to avoid contact with individuals affected with COVID-19 (74.3%) or AIDS (59.0%), as well as their family members (70.4% for COVID-19 and 47.9% for AIDS). About half of the participants agreed that affected persons could not only cause problems to their own family but also have adverse effects on others (59.6% and 55.6% for COVID-19, 56.9 and 47.0% for AIDS). The agreements with statements about perceived stigma were similar but slightly higher than those about personal stigma in both COVID-19 and AIDS. Participants' agreements with all statements regarding personal and perceived stigma attitudes between COVID-19 and AIDS were all statistically significant (*p* < 0.001). Participants obtained COVID-19-related information mainly from social media (91.3%) and newspaper or television (77.1%) during the epidemic, and 61.0% of them thought information from newspapers or television was the most reliable.

**Conclusion:** Several similarities and differences of people's attitude toward COVID-19 and AIDS were found. Avoidance, blame, and secondary discrimination to diagnosed persons and their surrounding persons were the main representations of COVID-19-related stigma. Stigma of COVID-19 had less moral link but more public panic. Experience from HIV-related stigma reduction and prevention can be applied to reduce COVID-19-related stigma.

## Introduction

Novel Coronavirus Disease 2019 (COVID-19) is an infectious disease caused by severe acute respiratory syndrome coronavirus 2 (SARS-COV-2). The disease spectrum caused by this virus ranges from asymptomatic, fever, cough, and fatigue to severe acute respiratory distress syndrome (ARDS), and even death (1). According to a report of 72,314 cases in China, 81% patients' symptom was sorted as mild, 14% were severe that need ventilation in an intensive care unit (ICU), and 5% were critical that had respiratory failure, septic shock, and/or multiple-organ dysfunction or failure (2). SARS-COV-2 spreads mainly via respiratory and closed contact (3) and is infectious during the latent period (4) which ranges from 2 to 14 days (median: 4–5 days) (5). As the strong infectivity (median *R*_0_ = 5.7, 95% CI: 3.8–8.9) and fast transmission of SARS-COV-2 (6), COVID-19 soon spread around the world. The WHO proclaimed COVID-19 as a public health emergency and designated it a pandemic on March 11, 2020 (7). There are several vaccines available for COVID-19 which could provide protection for those older than 16 to some extent (8). However, some variations of SARS-COV-2 have been detected globally and the efficacy of vaccines has absolute marked differences (9, 10).

Stigma was first proposed by Erving Goffman in 1963, which was defined as a “sign” or an attribute that reduces an individual's status in the eyes of society (11). It was also interpreted as a mark of shame and disapproval that result in a person apart from others (12). It refers to people's negative emotional experience of disease, including personal stigma and perceived stigma. Personal stigma is a process of stereotype, prejudice, and discrimination, while perceived stigma indicates that someone is approved of the public discrimination against the group (13). Extreme fear of a disease and self-defense may be related to stigma. Mental disorders, physical disability, and emerging infectious diseases have been reported with different degrees of stigma (14). Stigma has always been a major focus throughout the pandemic of an infectious disease (3). The impact of infectious disease stigma is no less than the disease itself. Not only does it influence the patients' quality of life and social ability, but it also affects the publics' attitude toward disease prevention, service delivery, medical resource allocation, and health policymaking (15). Isolation measures were taken during the outbreak of COVID-19, which effectively decreased the morbidity and mortality of COVID-19 but may increase stigma inversely (16). Some scholars pointed that compared with other regions, people resident in the infectious area were more likely to be prejudiced and discriminated (17). The fear of getting infected of COVID-19 and self-defense might contribute to stigmatizing attitude (18), and the stigma of this infectious disease may inversely lead to delayed help-seeking. COVID-19-related stigma may pose a serious threat to COVID-19 patients and survivors, as well as their families and surrounding people. Several incidents of stigmatization, even physical violence toward patients, survivors, and medical workers, have occurred during this pandemic all around the world (19). There were numerous studies investigating sleeping disorder, anxiety, depression, post-traumatic stress disorder, and other mental disorders related since the outbreak of COVID-19; however, few have focused on COVID-19-related stigma (20). Since there is no effective therapy toward COVID-19 so far, people's attitude to COVID-19 is worth investigating.

Acquired immunodeficiency syndrome (AIDS), caused by human immunodeficiency virus (HIV), is another kind of infectious disease transmitted mainly *via* unprotected sexual activity, contaminated blood transfusion, and contaminated needles and from mother to child during pregnancy (21). Numerous studies about AIDS and its stigma have been done (22), and several systematic reviews have been published (23, 24). Previous stigma-related studies on AIDS reported that HIV-positive individuals were more vulnerable to receive stigma from others, which usually contain health, moral, and racial dimensions and promoted stigma including intrapersonal, interpersonal, and social aspects (25). Both COVID-19 and AIDS are infectious diseases with no definite therapy, and suffering from COVID-19 or AIDS will cause a certain damage to both individuals and our society. Therefore, we try to learn COVID-19 stigma by comparing with AIDS stigma, as Logie thought that we can learn the experience of studying AIDS stigma and leverage the approaches used to reduce AIDS stigma to address COVID-19 stigma (26).

Hence, we conducted this study with the aims of (1) investigating publics' stigmatizing attitudes toward COVID-19 and (2) comparing publics' stigmatizing attitudes between COVID-19 and AIDS to find the similarities and differences. From this study, we hope to provide some theoretical basis for psychological intervention toward COVID-19 stigma and further policymaking.

## Materials and Methods

### Participants

Participants were recruited online, and inclusion criteria were (1) age ≥16, (2) can fully understand the informed consent and questionnaire, (3) willing to participate in the survey and can sign the informed consent online.

### Procedures and Materials

Data were obtained using the convenient sampling method through a WeChat-based questionnaire including demographic questionnaire, a stigma scale that was adopted from the Explanatory Model Interview Catalog-Community Stigma Scale (EMIC-CSS) (27), and Depression Stigma Scale (DSS) (28). Participants' demographic information such as gender, age, education, and occupation was collected through a demographic questionnaire. The 18-item stigma-related scale consists of personal stigma aspect and perceived stigma aspect with nine items separately (seen in Supplementary Table S1) and was used to measure participants' stigma attitudes toward COVID-19 and AIDS. A vignette-based survey method was used in this study. A hypothetical case diagnosed with COVID-19 and a case diagnosed with AIDS were listed separately, followed by 18 questions evaluating participants' personal and perceived stigma toward the hypothetical case. Participants were asked to choose their own answers from “strongly agree,” “agree,” “uncertain,” “disagree,” and “strongly disagree.” The vignettes and stigma-related questions were as follows.

Vignette of COVID-19: “Li Ming (pseudonym) has been living in Wuhan. After the outbreak of COVID-19, he consciously isolated himself at home and wore a mask when he went out occasionally. Li Ming recently had a fever, cough and other symptoms. He was diagnosed with new coronavirus pneumonia and has been hospitalized. Li Ming did not know he was infected with the virus until he has been diagnosed.”

Vignette of AIDS: “Zhang Yi (pseudonym) has been living in Wuhan. After the outbreak of the COVID-19, he consciously isolated himself at home and wore a mask when he went out occasionally. Zhang Yi recently had a fever, fatigue and other symptoms. He was diagnosed with AIDS and has been hospitalized. Zhang Yi did not know he was infected with HIV until he has been diagnosed.”

Public's personal stigma attitudes were measured by the following nine questions: (1) If I were him, I would prefer to keep people from knowing about my situation; (2) I'm not willing to provide home service (such as delivery) for him or visit his home; (3) I think that he was affected by the disease because of his carelessness; (4) I think that his situation will cause problems to his family; (5) I think that his situation will have an adverse effect on others; (6) I will look down on him; (7) I try to avoid contact with him, especially physical contact; (8) I try to avoid contact with his family; and (9) I will look down on his family because of his situation.

Public's perceived stigma attitudes were measured by replacing “I think/will…” with “Most people think/will…” of the above nine questions.

We also investigated the usual source that participants used to get the COVID-19-related knowledge during the epidemic to estimate the role of each medium in spreading information.

### Ethical Considerations

The study protocol was approved by the Ethics Committee of Second Xiangya Hospital, Central South University. Informed consents were listed on the first page of the questionnaire independently. Before answering questions, potential participants were asked to read informed consents carefully and determined whether they were willing to participate in this study. Those who click “yes” would obtain the whole questionnaire to complete, while others were displayed an end page of this study and appreciation.

### Statistical Analysis

Frequency and percentage were used to describe demographic data while percentage frequencies and 95% confidence interval (CI) were computed for stigma items. Categories of “strongly agree” and “agree” were merged into “agreement” for descriptions. A paired *T*-test was used to compare participants' stigmatizing attitudes between the two vignettes. All data analyses were conducted in SPSS 25.0, and *p* < 0.05 was considered as statistically significant.

## Results

### Demographic Information

In total, 19,355 questionnaires were collected and 5,341 were excluded after manual review. The screening principles were as follows: (1) <2 s to finish each item, (2) ≥2 questionnaires from the same IP (only the first one was retained), (3) obvious errors, e.g., a 17-year-old person chooses “married” in the marriage item. Finally, 13,994 participants (55.4% male) were included with the efficiency of 72.3%. The average age was (30.44 ± 9.63) (x ± s); 65.9% of the participants were from city. Over 54.3% participants were with the educated year longer than 12 years, and 13.3% were residents in Wuhan province during the epidemic. More demographic details are seen in Table 1.

**Table 1:** Demographic characteristics of participants (*N* = 13,994).

	***n***	**(%)**
Gender		
Male	7,757	55.4
Female	6,237	44.6
Age	30.4 ± 9.6	
Residence		
Countryside	4,765	34.1
City	9,229	65.9
Residence during the epidemic		
Hubei province	1,864	13.3
Other province in China except Hubei	12,017	85.9
Overseas	113	0.8
Education (years)		
≤9	901	6.4
≤12	5,352	38.3
≤16	6,273	44.8
>16	1,468	10.5
Marriage		
Single	5,968	42.6
Married	7,423	53.1
Others (divorced/widowed)	603	4.3
Income per year (thousand)		
≤50	5,815	41.6
60–100	5,256	37.6
110–190	2,091	14.9
≥200	832	5.9
Occupation		
Clinical staff	1,790	12.8
Civil servant	964	6.9
Employees	4,517	32.3
Medical students	1,017	7.3
Non-medical students	1,783	12.7
Self-employed	2,999	21.4
Others	924	6.6

### Personal Stigma Toward COVID-19 and AIDS

Participants' own attitudes toward COVID-19 and AIDS are presented in Table 2. Participants were most likely to agree to avoid contact either with people diagnosed with COVID-19 or with their family members, as 74.3% participants strongly agreed or agreed to avoid contact with people diagnosed with COVID-19 and 70.4% strongly agreed or agreed to avoid contact with their family, while 59.0 and 47.9% participants tended to avoid contact with individuals diagnosed with AIDS and their families. Participants' agreements with the above two statements between COVID-19 and AIDS were significantly different. The third highest agreed statement toward COVID-19 was “I think his situation will cause problems to his family” (59.6%), while 56.9% endorsed with the statement toward AIDS. There was also a high proportion of participants that thought that sufferers would have an adverse effect on others (55.6% for COVID-19 and 47.0% for AIDS). Endorsement with unwillingness to provide home service (such as delivery) or visit his home was 39.5% for COVID-19 and 35.1% for AIDS patients. Participants' agreement with keeping people from knowing their situation was 21.2% for COVID-19 and 38.4% for AIDS. Belief that suffering from COVID-19 or AIDS was patients' own fault was 23.0% for COVID-19 and 39.3% for AIDS. Agreement with the statement that they would look down upon the individuals with disease was 11.6% for COVID-19 and 17.8% for AIDS. Even 14.5% participants for COVID-19 and 17.8% for AIDS agreed that they would look down on patients' family because of the patients' situation. Participants' agreements with all of the above statements about their own attitudes between COVID-19 and AIDS were statistically significant (*p* < 0.001).

**Table 2:** Percentage and 95% CI of participants who “agree” or “strongly agree” with statements about their own attitudes toward the person described in the vignette (*N* = 13,994).

**Statements**	**COVID-19**		**AIDS**		***p* ^a^**
	***n***	**%**	***n***	**%**	
If I were him, I would prefer to keep people from knowing about my situation	2,968	21.2 (20.6–21.9)	5,368	38.4 (37.6–39.2)	<0.001
I will look down on him	1,627	11.6 (11.1–12.2)	2,485	17.8 (17.1–18.4)	<0.001
I think his situation was caused by his own fault	3,224	23.0 (22.3–23.7)	5,502	39.3 (38.5–40.1)	<0.001
I think his situation will cause problems to his family	8,340	59.6 (58.8–60.4)	7,962	56.9 (56.1–57.7)	<0.001
I will look down on his family because of his situation	2,035	14.5 (14.0–15.1)	2,403	17.2 (16.5–17.8)	<0.001
I think his situation will have an adverse effect on others	7,779	55.6 (54.8–56.4)	6,704	47.0 (46.2–47.8)	<0.001
I will try to avoid contact with him, especially physical contact	10,401	74.3 (73.6–75.0)	8,254	59.0 (58.2–59.8)	<0.001
I will try to avoid contact with his family	9,853	70.4 (69.7–71.2)	6,575	47.9 (47.1–48.7)	<0.001
I am not willing to provide home service (such as delivery) for him or visit his home	5,523	39.5 (38.7–40.3)	4,912	35.1 (34.3–35.9)	<0.001

a *The p value of paired-t test*.

### Perceived Stigma Toward COVID-19 and AIDS

Participants' agreements with statements about public attitudes are described in Table 3. Over 70% participants tended to agree that others would try to avoid contact with COVID-19 individuals (76.4%) and their family (74.3%), while the proportions of agreements in vignette of AIDS were 61.3% for individuals and 49.8% for their family. In the COVID-19 vignette, most participants agreed that the patients would cause problems to their family (64.0%) and have side effects on others (59.2%), while in the AIDS vignette, the percentages of agreement were 59.9 and 53.5%, separately. Belief that most people were unwilling to provide home service (such as delivery) for the individual or visit his home was 51.6% for COVID-19 vignette, and 43.2% for AIDS vignette. More detailed information is described in Table 3. Participants' agreements with all of the above statements about most other people's attitudes between COVID-19 and AIDS were also statistically significant (*p* < 0.001).

**Table 3:** Percentage and 95% CI of participants who “agree” or “strongly agree” with statements about most others people's attitudes toward the person described in the vignette (*N* = 13,994).

**Statements**	**COVID-19**		**AIDS**		***p* ^a^**
	***n***	**%**	***n***	**%**	
Most people think he would prefer to keep people from knowing about his situation	3,382	24.2 (23.4–24.9)	6,389	45.7 (44.8–46.5)	<0.001
Most people will look down on him	2,343	16.7 (16.1–17.4)	4,431	31.7 (30.9–32.4)	<0.001
Most people think that his situation was caused by his own fault	5,527	39.5 (38.7–40.3)	7,597	54.3 (53.5–55.1)	<0.001
Most people think that his situation will cause problems to his family	8,951	64.0 (63.2–64.8)	8,379	59.9 (59.1–60.7)	<0.001
Most people will look down on his family because of his situation	2,608	18.6 (18.0–19.3)	3,442	24.6 (23.9–25.3)	<0.001
Most people think that his situation will have an adverse effect on others	8,288	59.2 (58.4–60.0)	7,388	53.5 (52.7–54.3)	<0.001
Most people try to avoid contact with him, especially physical contact	10,688	76.4 (75.7–77.1)	8,579	61.3 (60.5–62.1)	<0.001
Most people try to avoid contact with his family	10,399	74.3 (73.6–75.0)	6,976	49.8 (49.0–50.7)	<0.001
Most people aren't willing to provide home service (such as delivery) for him or visit his home	7,224	51.6 (50.8–52.5)	6,050	43.2 (42.4–44.1)	<0.001

a *The p value of paired-t test*.

### Usual Source to Get COVID-19-Related Knowledge

Participants received COVID-19-related information was mainly from social media (91.3%), newspaper or television (77.1%), initiative network inquiring (53.7%), and community publicity (32.6%) during the epidemic. Among that, over 60% of participants obtained most of the information from social media while 61.0% participants regarded the newspaper and television as the most reliable resource; details are listed in Table 4.

**Table 4:** Usual source that participants got COVID-19 related knowledge during the epidemic (*n*, %).

	**Newspapers/TV**	**Social media**	**Initiative network inquiring**	**Community publicity**
Channels to get epidemic information	10,786 (77.1)	12,777 (91.3)	7,514 (53.7)	4,556 (32.6)
Channel to obtain most of the information	3,169 (22.6)	8,441 (60.3)	1,860 (13.3)	524 (3.7)
The most reliable channel	8,535 (61.0)	3,685 (26.3)	1,185 (8.5)	589 (4.2)

## Discussion

This study explored publics' stigmatizing attitudes toward COVID-19 during the epidemic and compared it with stigmatizing attitudes toward AIDS. The results showed that for COVID-19 beliefs about avoiding contact with individuals with COVID-19 and their families, individuals with COVID-19 would cause problems to or have an adverse effect on their families and others were much higher than other statements either in personal stigma or perceived stigma. For perceived stigma, unwillingness to provide home service or visit the home of individuals with COVID-19 was also among the highly agreed statements. Participants' highly agreed statements toward AIDS were similar with COVID-19 but had a slightly lower proportion, which were significantly different.

In the personal stigma dimension of COVID-19, people tend to keep distance with individuals diagnosed with COVID-19, which is in accordance with the study by Sing Lee (29); they found that social distance might be related to severe acute respiratory syndrome (SARS) stigma. As close contact was one of the common transmission methods of COVID-19 (30), the Chinese government took several effective measures to stop people from contacting each other immediately after the outbreak of COVID-19, such as isolation, social distancing, community containment, and travel restriction (3). These policies effectively lower the transmission rate of COVID-19 but may produce stigmatization at the same time. Isolated individuals are more likely to suffer from stigmatization and social rejection (31). Some researchers claimed that stigma might negatively affect those with COVID-19 as well as their families, friends, caregivers, and communities (3). They might be experiencing “secondary” or “associative” stigma (32). There were several reports about COVID-19-related stigma to healthcare providers. In this study, numerous participants reported unwillingness to provide home service, which is similar to the existing views that the stigmatized group may experience stigmatizing behaviors such as isolation, refusal to provide service, and bullying (33). A relieving discovery was the low agreements about the statement of “I will look down on him or his family.” This may be because COVID-19 spreads mainly through respiratory, and stigmatization against an individual is relatively lower than avoiding physical contact. It should be noted that in this study, 21.2% participants tended to keep it a secret if they were diagnosed with COVID-19, which can seriously expand the transmission and mislead the government into making wrong decisions about the epidemic and increase the difficulty of epidemic control.

In perceived stigma dimension, agreements with statements about COVID-19 were roughly similar to the statements described in personal stigma, but the proportion of each statement was slightly higher. That might be because people tend to answer the questions in an acceptable way to cater to public requirements (34).

AIDS stigma has been investigated by many scholars. In this survey, we found a number of similarities and differences between AIDS and COVID-19 stigma. A large proportion of participants were inclined to agree with avoidance of patients and their surrounding people and hold the opinion that patients would encumber others. This might be due to the similarity of infectivity and the psychological perspective that the negative emotions aroused by the two diseases generate similar patterns of stigmatization (35). Participants were more likely to keep it a secret if they suffered from AIDS compared with COVID-19 for both personal and perceived stigma. Policy and moral condemnation may contribute to this difference. The Chinese government has already made some punishment policies to reduce the incidence of concealment and omission during the pandemic of COVID-19. Ways of transmission are quite different between these two diseases—primarily sexual and blood-to-blood for AIDS and primarily droplet transmission for COVID-19 (3). Hence, AIDS is usually conceptually linked to morality and equated with sexual promiscuity, homosexuality, drug abuse, and personal irresponsibility (36), while people with COVID-19 are less morally condemned. A higher proportion of participants thought that individuals with AIDS were more likely to be responsible for their situation and be looked down upon, but they may cause less problem to others compared with people with COVID-19. This might also relate to the different transmission methods of the two diseases and may indicate that stigma of COVID-19 had less moral link but more public panic.

Public response is closely related to the information they get and media report. Media report can powerfully influence public attitudes. Social media and newspapers/TV are the main usual source for the public to get information about COVID-19. Social media could affect people's attitudes of risk perception while legacy media could affect public perceptions of protective behaviors. When the COVID-19 crisis was reported on TV or social media, some information might be misunderstood. Misinformation and rumors may produce public anxiety and panic and lead to a series of related behaviors such as prohibiting medical workers from going back home for fear of being infected. These media platforms are supposed to enhance public awareness without increasing fear and panic (37). Hence, measures should be taken to ensure the correct dissemination of information and reduce rumors during and after the pandemic.

In the present era, increasing our ability to reduce the stigmatization associated with emerging infectious diseases is required in controlling such diseases. A variety of methods have been taken with the attempt to reduce stigmatization associated with AIDS, such as basic public education about AIDS, publicized symbolic acts by public leaders or famous people, media campaigns, and designation of December 1 as World AIDS Day. These efforts have achieved some success (38). Our study showed many similarities between COVID-19-related stigma and AIDS-related stigma; therefore, we could use the anti-AIDS-related stigma approaches to reduce COVID-19 stigma. Anti-stigma approaches toward mental disorders could also be considered. A pilot study on an anti-stigma course toward mental disorders, which consisted of three components, namely, social contact, role-playing, and critical reflection strategies, showed that participants' stigma attitudes were significantly reduced after the 18-week anti-stigma course (39). Another study examining the potential impact of an anti-stigma intervention on help-seeking attitudes, which included education about depression, information about help-seeking, and contact with a person with lived experience, showed improvements in help-seeking attitudes (40). Our data indicate that providing accurate COVID-19-related information through social media and newspapers/TV may be effective as these are the main sources they used to get COVID-19-related information. Public education may be another useful approach, and the above-mentioned participants' highly agreed statements should be taken into consideration.

To our knowledge, this is the first study to compare COVID-19 related stigma with AIDS related stigma. This survey has some limitations that need to be noticed. Firstly, convenience sampling method was used to collect data from the public by anonymous internet questionnaires, which might be the major limitation. Compared with random sampling method, convenience sampling method might easily lead to sampling error and bias, so that our respondents cannot represent well the population level. The sampling error may lead to inaccuracy conclusions. However, we tried to get as large a sample size as we can and be more cautious with our conclusions in order to avoid inaccuracy conclusions. Secondly, this was a cross-sectional study conducted during the pandemic, which can only reflect participants' attitude toward COVID-19 during the outbreak in China. Public's attitudes toward COVID-19 may change as we know more about this disease; we now are conducting a follow-up study to further investigate it. Thirdly, COVID-19 and AIDS are both infectious diseases but differ in transmission. There is no definite answer to whether the stigmatizations between these two diseases are completely comparable. A previous study has compared Chinese health professionals' attitudes toward patients with AIDS vs. patients with hepatitis B and found that health professionals had negative biases against AIDS patients and less willingness to interact with AIDS patients compared with hepatitis B patients (41), which indicates that stigmatizing attitudes toward COVID-19 and AIDS may be comparable to some extent. Logie also pointed out that we can use the experience of studying AIDS-related stigma and the approaches used in order to explore COVID-19-related stigma (26). Fourthly, we did not compare publics' stigma toward COVID-19 with stigma against non-communicable diseases such as mental disorders in this study. More efforts will be made to the comparison mentioned above in our future study. Another limitation is that the scale we used was adopted from the Explanatory Model Interview Catalog-Community Stigma Scale, which may not evaluate all aspects of COVID-19-related stigma. Hence, we just illustrate COVID-19-related stigma by describing the proportion of agreement with statements of the listed stigma-related items. Further non-convenience sampling and longitudinal study should be done to investigate more aspects of COVID-19-related stigma.

## Conclusions

Several similarities and differences in people's attitude toward COVID-19 and AIDS were found in this cross-sectional study. Avoidance, blame, and secondary discrimination to diagnosed persons and their surrounding persons were the main representations of stigma. Stigma of COVID-19 had less moral link but more public panic. Social media, television, and newspapers played a cardinal role in dissemination during the pandemic. Experience from AIDS-related stigma reduction and prevention can be applied to reduce COVID-19-related stigma. Social media, television, and newspapers should be made the best use, and the abovementioned highly agreed statements should be taken into consideration in further anti-stigma campaigns.

## Data Availability Statement

The raw data supporting the conclusions of this article will be made available by the authors, without undue reservation.

## Ethics Statement

The study protocol was approved by the Ethics Committee of Second Xiangya Hospital, Central South University. Informed consents were listed on the first page of the questionnaire independently. Before answering questions, potential participants were asked to read informed consents carefully and determined whether they were willing to participate in this study. Those who click yes would obtain the whole questionnaire to complete while others were displayed an end page of this study and appreciation.

## Author Contributions

This study was conceptualized by TL, SC, YaL, and XW. The database was organized by YZ, YiW, YM, and YuL. Data analysis was done by ML and YuW. The manuscript with inputs was drafted by ML and YH. Reviewed by TL, SC, and JL. All authors read and approved the final manuscript.

## Funding

This study was supported by the National Key R&D Program of China (2017YFC1310400) and the Natural Science Foundation of Hunan Province (2020JJ4795).

## Conflict of Interest

The authors declare that the research was conducted in the absence of any commercial or financial relationships that could be construed as a potential conflict of interest.

## Publisher's Note

All claims expressed in this article are solely those of the authors and do not necessarily represent those of their affiliated organizations, or those of the publisher, the editors and the reviewers. Any product that may be evaluated in this article, or claim that may be made by its manufacturer, is not guaranteed or endorsed by the publisher.
