# Corrigendum: A Comparison of COVID-19 Stigma and AIDS Stigma During the COVID-19 Pandemic: A Cross-Sectional Study in China

**DOI:** 10.3389/fpsyt.2022.856704

**Published:** 2022-02-11

**Authors:** Manyun Li, Jiang Long, Xuyi Wang, Yanhui Liao, Yueheng Liu, Yuzhu Hao, Qiuxia Wu, Yanan Zhou, Yingying Wang, Yunfei Wang, Qianjin Wang, Yuejiao Ma, Shubao Chen, Tieqiao Liu

**Affiliations:** ^1^Department of Psychiatry, and National Clinical Research Center for Mental Disorders, The Second Xiangya Hospital of Central South University, Changsha, China; ^2^Shanghai Mental Health Center, Shanghai Jiao Tong University School of Medicine, Shanghai, China; ^3^Department of Psychiatry, Sir Run Run Shaw Hospital, School of Medicine, Zhejiang University, Hangzhou, China; ^4^Department of Psychiatry, Hunan Brain Hospital (Hunan Second People's Hospital), Changsha, China

**Keywords:** COVID-19, AIDS, stigma, physical avoidance, public panic

In the published article, there was an error in affiliation **1**–**5**. Instead of “^1^ National Clinical Research Center for Mental Disorders, Changsha, China, ^2^ Department of Psychiatry, The Second Xiangya Hospital of Central South University, Changsha, China ^3^ Shanghai Mental Health Center, Shanghai Jiao Tong University School of Medicine, Shanghai, China, ^4^ Department of Psychiatry, Sir Run Run Shaw Hospital, School of Medicine, Zhejiang University, Hangzhou, China, ^5^ Department of Psychiatry, Hunan Brain Hospital (Hunan Second People's Hospital), Changsha, China,” it should be “^1^ Department of Psychiatry, and National Clinical Research Center for Mental Disorders, The Second Xiangya Hospital of Central South University, Changsha, China, ^2^ Shanghai Mental Health Center, Shanghai Jiao Tong University School of Medicine, Shanghai, China, ^3^ Department of Psychiatry, Sir Run Run Shaw Hospital, School of Medicine, Zhejiang University, Hangzhou, China, ^4^ Department of Psychiatry, Hunan Brain Hospital (Hunan Second People's Hospital), Changsha, China.”

The authors apologize for this error and state that this does not change the scientific conclusions of the article in any way. The original article has been updated.

## Publisher's Note

All claims expressed in this article are solely those of the authors and do not necessarily represent those of their affiliated organizations, or those of the publisher, the editors and the reviewers. Any product that may be evaluated in this article, or claim that may be made by its manufacturer, is not guaranteed or endorsed by the publisher.

