# Supplementary material for: A Comparison of COVID-19 Stigma and AIDS Stigma During the COVID-19 Pandemic: A Cross-Sectional Study in China
Source: Front Psychiatry. 2021 Dec 1;12:782501. doi: 10.3389/fpsyt.2021.782501 (PMC8671734; doi:10.3389/fpsyt.2021.782501)
Supplement: Supplementary file 1 [file Table_1.pdf]

SUPPLEMENTAL DATA

Table S1. Detailed items of stigma scale

Items
1.Personal stigma items
If I were him, I would prefer to keep people from knowing about my situation
I will look down on him
I think his situation was caused by his own fault
I think his situation will cause problems to his family
I will look down on his family because of his situation
I think his situation will have an adverse effect on others
I will try to avoid contact with him, especially physical contact
I will try to avoid contact with his family
I am not willing to provide home service (such as delivery) for him or visit his home
2.Perceived stigma items
Most people think he would prefer to keep people from knowing about his situation
Most people will look down on him
Most people think that his situation was caused by his own fault
Most people think that his situation will cause problems to his family
Most people will look down on his family because of his situation
Most people think that his situation will have an adverse effect on others
Most people try to avoid contact with him, especially physical contact
Most people try to avoid contact with his family
Most people aren't willing to provide home service (such as delivery) for him or visit his home
